# Isolation and Characterization of Insecticidal Toxins from the Venom of the North African Scorpion, *Buthacus leptochelys*

**DOI:** 10.3390/toxins11040236

**Published:** 2019-04-25

**Authors:** Yusuke Yoshimoto, Masahiro Miyashita, Mohammed Abdel-Wahab, Moustafa Sarhan, Yoshiaki Nakagawa, Hisashi Miyagawa

**Affiliations:** 1 Division of Applied Life Sciences, Graduate School of Agriculture, Kyoto University, Kyoto 606-8502, Japan; yoshimoto.yusuke.22v@st.kyoto-u.ac.jp (Y.Y.); naka@kais.kyoto-u.ac.jp (Y.N.); miyagawa@kais.kyoto-u.ac.jp (H.M.); 2 Zoology Department, Al-Azhar University, Assuit 71524, Egypt; manatee74@yahoo.com (M.A.-W.); moustafar@yahoo.com (M.S.)

**Keywords:** scorpion venom, insecticidal peptide, mass spectrometric analysis, de novo sequencing

## Abstract

Various bioactive peptides have been identified in scorpion venom, but there are many scorpion species whose venom has not been investigated. In this study, we characterized venom components of the North African scorpion, *Buthacus leptochelys*, by mass spectrometric analysis and evaluated their insect toxicity. This is the first report of chemical and biological characterization of the *B. leptochelys* venom. LC/MS analysis detected at least 148 components in the venom. We isolated four peptides that show insect toxicity (Bl-1, Bl-2, Bl-3, and Bl-4) through bioassay-guided HPLC fractionation. These toxins were found to be similar to scorpion α- and β-toxins based on their N-terminal sequences. Among them, the complete primary structure of Bl-1 was determined by combination of Edman degradation and MS/MS analysis. Bl-1 is composed of 67 amino acid residues and crosslinked with four disulfide bonds. Since Bl-1 shares high sequence similarity with α-like toxins, it is likely that it acts on Na^+^ channels of both insects and mammals.

## 1. Introduction

Scorpions are the oldest arachnids and can be traced back to the Silurian period [1,2]. Currently, over 2400 scorpion species are widely distributed on all continents except Antarctica [3,4]. Scorpions have adapted to different environments such as deserts, forests, grasslands, and caves because they can use toxic components in their venom to effectively capture prey and protect themselves from predators. Scorpion venom contains inorganic salts, amino acids, nucleic acids, peptides, and proteins, and some peptides show anti-insect and/or anti-mammal activities [5]. 

Scorpion peptides are structurally classified into two groups: disulfide bridge-containing peptides (DBPs) and non-disulfide bridge-containing peptides (NDBPs) [6,7]. Many DBPs specifically interact with neuronal ion channels, which are further classified into four families based on their targeting ion channels (Na^+^, K^+^, Cl^−^, and Ca^2+^ channels) [8,9]. Sodium channel-specific toxins are long-chain peptides composed of 60–76 amino acid residues cross-linked with four disulfide bridges [10,11,12]. These toxins are divided into α- and β-toxins. The α-toxins inactivate Na^+^ current by binding to the receptor site 3, whereas the β-toxins modify the voltage-dependent activation of sodium channels by binding to the receptor site 4 [13,14]. Potassium channel-specific toxins are composed of 20–70 amino acid residues cross-linked with three or four disulfide bridges. Most of these are short-chain peptides with fewer than 40 amino acid residues [15,16]. These toxins are classified into seven families, α-, β-, γ-, κ-, δ-, λ-, and ε-KTx, according to their sequence similarity and disulfide-bonding pattern [17,18]. Chloride ion channel-specific toxins are composed of fewer than 40 amino acid residues and contain four disulfide bonds [19,20]. Some toxins specifically act on calcium ion channels, which have varied structures and action sites [8,21]. On the other hand, many NDBPs adopt an amphipathic α-helical structure that can disrupt the cellular membrane structure to show antimicrobial and/or hemolytic activity [22].

These scorpion peptides have been identified from various scorpion species, but venoms of minor species remain largely unstudied. About 930 scorpion species inhabit Africa, most of which belong to the Buthidae family [23]. In this family, *Leiurus quinquestriatus* has been intensively studied because it contains medically important toxins [24]. The venom of the species of the genus *Buthacus* in the Buthidae family has been poorly investigated, and only two insecticidal toxins were isolated from this genus: Bu1 from *B. macrocentrus* and BaIT2 from *B. arenicola* [25,26]. In this study, we evaluated the insect toxicity of the venom of *B. leptochelys* and isolated four insecticidal peptides from the venom using a bioassay-guided HPLC fractionation approach. Finally, one of the insecticidal components in the *B. leptochelys* venom was identified by the combination of Edman degradation and MS/MS analysis.

## 2. Results and Discussion

### 2.1. Characterization of the B. leptochelys Venom

The venom showed significant toxicity against crickets (*A. domesticus*) with an LD_50_ value of 30 ng/mg body weight. This is 3-fold more potent than that of *Isometrus maculatus,* which has moderately toxic venom [27], but 1.5-fold less potent than that of *Tityus serrulatus,* which has highly toxic venom, although a different cricket species was used in the latter case [28]. 

**Figure 1:**
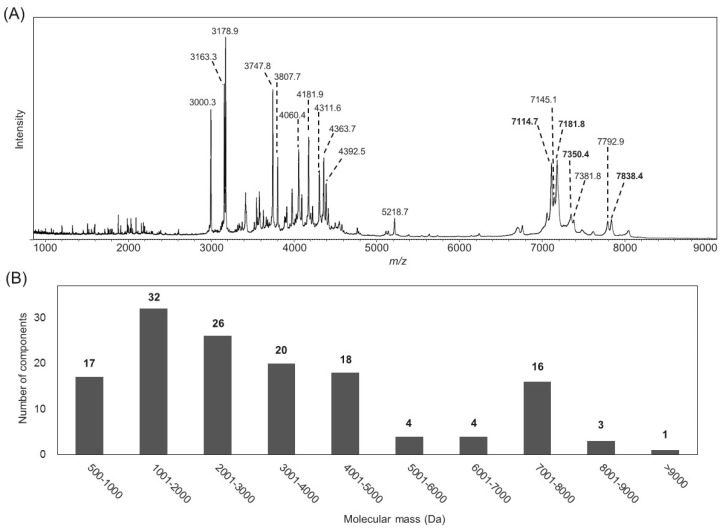
Components analysis of the *B. leptochelys* venom. (**A**) MALDI-TOF mass spectrum of the venom. Numbers shown in bold indicate the peptides isolated in this study. (**B**) Distribution of molecular masses of venom components detected by LC/MS analysis.

The *B. leptochelys* venom was then subjected to mass spectrometric analysis using both MALDI-TOF MS and LC/MS to examine the molecular mass distribution of all the venom components (Figure 1). A total of 148 components, which are likely to be peptides, were detected in a molecular mass ranging from 500 to 12,000 Da (Table S1). About 80% of the components were detected in the range below 5000 Da. Regarding the components with molecular masses over 5000 Da, the number of those with molecular masses ranging from 7001–8000 Da is relatively high. These peptides are likely to be long-chain scorpion peptides, which could mainly contribute to the insect toxicity of the *B. leptochelys* venom as reported for other Buthidae scorpions [29,30].

### 2.2. Purification of Insecticidal Peptides

**Figure 2:**
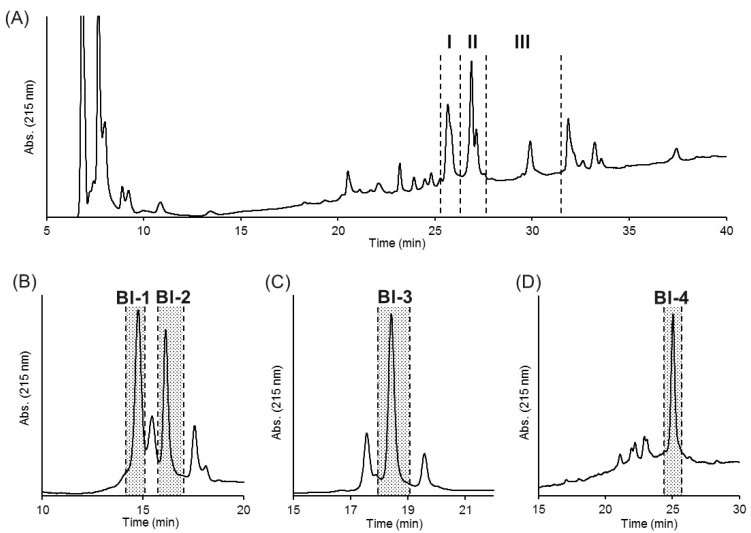
Isolation of the insecticidal peptides from the *B. leptochelys* venom. Separation of the crude venom on a C_4_ HPLC column (**A**). Separation of fractions I (**B**), II (**C**), and III (**D**) on a C_18_ HPLC column.

The crude venom dissolved in distilled water was separated on a C_4_ HPLC column (Figure 2A). Several fractions obtained based on the major chromatographic peaks were tested for insect toxicity using crickets. In this study, each fraction was injected at a dose equivalent to 160 ng venom/mg body weight, which is five times higher than the LD_50_ value, to specify peptides primarily responsible for the insect toxicity of the venom. Three fractions (I–III) were found to be highly toxic, and fraction I showed the strongest toxicity, which induced death or paralysis 48 h after injection in all insects tested. Fractions II and III showed relatively weak toxicity that induced transient paralysis in all insects tested. Differences in the insect toxicity between fractions could be attributed to the number and/or amount of active components in each fraction as well as to their intrinsic activity. These fractions were further separated on a C_18_ HPLC column to obtain single components. Four components (named Bl-1, 2, 3, and 4; Figure 2B–D) showed toxicity, and their monoisotopic molecular masses were determined as 7107.2, 7343.9, 7173.8, and 7828.1 Da, respectively (Figure S1). The N-terminal sequences of these peptides were determined by Edman method as shown in Table 1. 

A BLAST search revealed that Bl-1 is similar to α-like insect and mammal toxins such as Lqh3 from *Leiurus quinquestriatus hebraeus* [31] and Bom3 from *Buthus occitanus mardochei* [32]. Bl-2 is similar to α-mammalian toxins such as Lqq5 from *L. quinquestriatus quinquestriatus* [33] and AaH2 from *Androctonus australis* Hector [34]. Bl-3 is similar to α-insect and mammalian toxins such as OD1 from *Odontobuthus doriae* [35] and Bu-1 from *Buthacus macrocentrus* [25]. Bl-4 is similar to β-insect toxins such as AaHIT1 from *A. australis* Hector [36] and LqqIT1 from *L. quinquestriatus quinquestriatus* [37]. This suggests that the four insecticidal peptides isolated from the *B. leptochelys* venom may act on the insect Na^+^ channel, although the selectivity of their action between mammals and insects may vary among peptides. The α- and β-toxins have been identified exclusively from the venom of the Buthidae scorpions, but the ratio between the number of α- and β-toxins in the venom is known to differ by species. For example, 9 α- and 12 β-toxins were identified from the venom gland transcriptome of *Lychas mucronatus* [38], whereas 1 α- and 12 β-toxin sequences were identified from the *I. maculatus* transcriptome [39]. In addition, only β-toxins were isolated as an insecticidal neurotoxin from the *I. maculatus* venom [40,41]. The fact that the insecticidal activity of *B. leptochelys* venom is relatively higher than that of *I. maculatus* may be attributed to the existence of multiple α-toxins.

### 2.3. Primary Structure of Bl-1

**Figure 3:**
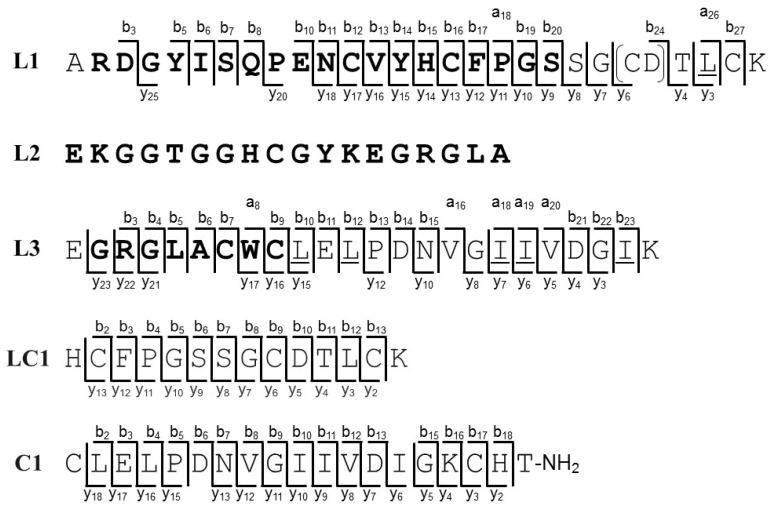
Amino acid seqences of digested fragments. Amino acid residues shown in bold were determined by Edman degradation. The MS/MS fragment ions that were used for sequence determination are shown. [CD] in the sequence of L1 indicates CD or DC. For L2, only the sequence determinated by Edman degradation is shown. Leu and Ile residues that are underlined were determined by MS/MS analysis inder the HE-CID conditon.

**Figure 4:**
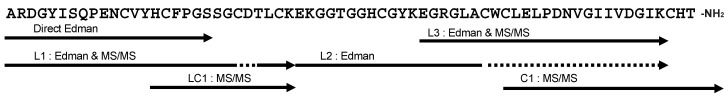
Primary structure of Bl-1. Solid lines indicate that the sequence was completely identified. Dashed lines show that the sequence was not or partically identified.

Bl-1 was further subjected to sequencing analysis to obtain its complete primary structure because it showed the most significant insect toxicity in this study. A 472 Da mass shift after carboxymethylation of Bl-1 is indicative of the presence of eight Cys residues (59 Da × 8) that form four disulfide bridges (Figure S2). Bl-1 was digested with endoproteinase Lys-C, and the resulting peptide fragments were purified by HPLC (Figure S3). The sequence of three fragments (L1, L2, and L3 with molecular masses of 3282.3, 3918.9, and 2686.3 Da, respectively) were determined by Edman and/or MS/MS sequencing analysis (Figure S4). Discrimination between Leu and Ile at several positions in the fragments during MS/MS analysis was achieved based on the side-chain fragmentation observed under HE-CID conditions in which the occurrence of key fragment ions (*d*-ions) allowed for its assignment (Figure S5). The sequences of L1 and L3 were determined as ARDGYISQPENCVYHCFPGSSG(CD/DC)TLCK and EGRGLACWCLELPDNVGIIVDIGK, respectively, by combination of Edman and MS/MS sequencing analysis (Figure 3). The N-terminal sequence of L2 was also determined as EKGGTGGHCGYKEGRGLA by Edman analysis, but other sequence information was not obtained by MS/MS analysis due to its large molecular mass. To assign the undefined sequence of cysteine and aspartic acid residues (CD or DC) in L1, Bl-1 was sequentially digested with Lys-C and chymotrypsin (Figure S3). The short fragment LC1 (molecular mass of 1628.6 Da) consisting of the C-terminal half of L1 was subjected to MS/MS analysis, and its sequence was determined as HCFPGSSGCDTLCK (Figure 3 and Figure S6). Moreover, carboxymethylated Bl-1 was digested with chymotrypsin, and the peptide fragment C1 (molecular mass of 2154.1 Da) was purified by HPLC (Figure S3). MS/MS analysis revealed the sequence of C1 as CLELPDNVGIIVDIGKCHT-NH_2_ by considering the sequence of L3 (Figure 3 and Figure S6). Since C1 has the amidated C-terminus, it was assigned as the C-terminal end of Bl-1 (Figure 4). The fragment C2 (molecular mass of 5444.2) was also detected by LC/MS analysis, which confirms the connection between L1 and L2 based on its molecular mass and partially determined sequence (Figure S7). Finally, the complete primary structure of Bl-1 was successfully determined by integrating all the information obtained above (Figure 4). 

### 2.4. Sequence Comparison

**Figure 5:**
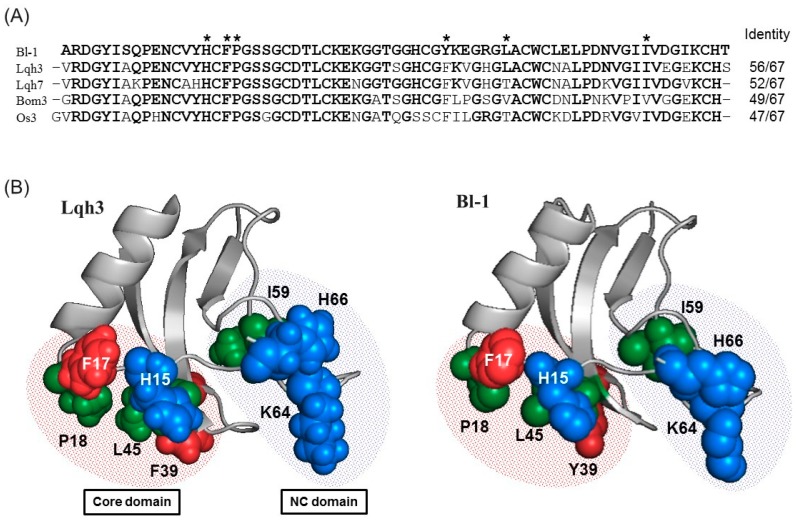
Comparison of the structure of Bl-1 with other toxins. (**A**) Multiple sequence alignment of Bl-1 with similar peptides found in the database. Asterisks indicate residues important for binding to insect sodium channels. (**B**) Three-dimensional structure of Lqh3 in PDB (ID: 1FH3) and Bl-1 constructed by homology modelings.

A BLAST search for a full sequence of Bl-1 revealed that the peptide is similar to scorpion peptides classified as an α-like toxin, which can modulate both insect and mammalian Na^+^ channels as described above (Figure 5A). This suggests that Bl-1 also shows toxicity against mammals, although mammal toxicity could not be evaluated due to the limited amount of the purified sample. Among the α-like toxins, the structure-activity relationship was comprehensively investigated for Lqh3 [42]. This study revealed that two distinct domains are particularly important for its binding to insect Na^+^ channels. One (Core-domain) consists of three residues (His15, Phe17, and Pro18) preceding the α-helix and two residues (Phe39 and Leu45) in the β-strands. The other (NC-domain) is constituted by the C-terminal region, where Ile59, Lys64, and His66 contribute the activity (Figure 5B). These residues are also observed in Bl-1, except for Phe39. Since the aromatic ring of Phe39 is important for the activity, the substitution of Phe39 with Tyr in the case of Bl-1 may not affect the activity. To further confirm the structural similarity between Lqh3 and Bl-1, a three-dimensional structure of Bl-1 was constructed by homology modeling using Lqh3 as a template (Figure 5B). As expected, positions of all amino acid residues important for expression of full activity were almost identical between Lqh3 and Bl-1. This suggests that Bl-1 exerts its insect toxicity through the same mechanism as α-like toxins such as Lqh3.

## 3. Conclusions

We characterized venom components of the North African scorpion, *B. leptochelys,* by mass spectrometric analysis and isolated the insecticidal peptides by the bioassay-guided fractionation approach. To our knowledge, this is the first report of the chemical and biological characterization of the *B. leptochelys* venom. Mass spectrometric analysis revealed that the venom components are mainly composed of two distinct groups based on the molecular mass ranges: one from 3000–5000 Da and the other from 7000–8000 Da, which is commonly observed for Buthidae scorpions. N-terminal sequences of four insecticidal peptides (Bl-1, Bl-2, Bl-3, and Bl-4) isolated from the *B. leptochelys* venom indicated that they are long-chain toxins that could specifically or non-specifically act on insect Na^+^ channels. Among them, the primary structure of Bl-1 was completely determined to be an α-like toxin, which is likely to act on both insect and mammal Na^+^ channels, by combination of Edman and MS/MS sequencing analysis. Insect toxicity is the common biological characteristic of scorpion venom, but the structure of insecticidal toxins and their combinations are diverse and complex among scorpion species. The results obtained in this study will provide a clue to understanding the synergistic role of α- and β-toxins in insecticidal activity in Buthidae scorpion venom.

## 4. Materials and Methods 

### 4.1. Collection of Venom

Scorpions *B. leptochelys* were collected at the Western Mediterranean coastal desert of Marsa Matruh in Egypt. The venom was collected in a microtube by squeezing the venom glands using fine forceps and dissolving it in distilled water. The crude venom was centrifuged at 14,000 rpm for 10 min at 4 °C. The supernatants were pooled, lyophilized, and stored at −80 °C.

### 4.2. Bioassay

Insect toxicity was tested by injection of 1–2 μL sample solutions in distilled water into the abdominal cavity of crickets (*Acheta domesticus*, 50 ± 5 mg body weight). Distilled water was injected as a negative control. Several doses of the venom were injected, and ten animals were used for each dose. For evaluation of each HPLC fraction, six animals were used. The number of paralyzed or dead animals were counted 48 h after injection. The dose required to induce 50% mortality (LD_50_) was calculated by statistical software GraphPad Prism 4 (GraphPad Software, San Diego, CA, USA). The research using experimental animals was approved by the Animal Experimentation Committee at Kyoto University (Permission number: 30-8; date of approval: 1 April 2018).

### 4.3. Mass Spectrometric Analysis

LC/MS and LC/MS^n^ measurements were carried out in the positive mode on an LCMS IT-TOF (Shimadzu, Kyoto, Japan) equipped with an electrospray ion source. HPLC separation was carried out on a reversed-phase C_18_ column (TSK-GEL, 1.0 mm ID × 150 mm, TOSOH, Tokyo, Japan). The column was eluted using a linear gradient from 5 to 60% solvent B (0.1% formic acid in acetonitrile) in solvent A (0.1% formic acid in water) for 110 min at a flow rate of 0.05 mL/min. The mass scale was externally calibrated using sodium trifluoroacetate cluster ions. Spectra were obtained over a mass range from *m/z* 400 to 2000, and the multiply charged molecular ions were manually deconvoluted to obtain molecular masses. The monoisotopic *m/z* values in each multiply charged ion were used for deconvolution. 

MALDI-TOF/TOF MS measurements were carried out on an Autoflex III smart beam (Bruker Daltonics, Billerica, MA, USA) with a nitrogen pulsed laser (337 nm). Samples were dissolved in 0.1% TFA in 50% acetonitrile/water and mixed with a matrix solution containing 10 mg/mL of α-cyano-4-hydroxycinnamic acid (CHCA) in acetone. An aliquot (0.5 μL) of matrix/acetone solution was spotted onto the MALDI sample target to generate a thin layer of matrix crystal. Then, 1 μL of the matrix/0.1% TFA in 50% acetonitrile/water solution was spotted onto the thin layer and then dried at room temperature. External calibration of the mass scale was carried out using the molecular masses of the known peptides. Interpretation of the MS/MS spectra was conducted manually with the help of the open-source software mMass [43].

### 4.4. HPLC Purification

The crude venom (2.0 mg) was dissolved in distilled water and separated by HPLC on a reversed-phase C_4_ column (Protein C_4_, 10 mm ID × 250 mm, Grace Vydac, Deerfield, IL, USA). The column was eluted using a linear gradient from 15 to 60% solvent D (0.08% TFA in acetonitrile) in solvent C (0.1% TFA in water) for 50 min at a flow rate of 2 mL/min. Elution was monitored by UV absorbance at 215 and 280 nm. Seven fractions were obtained based on the major chromatographic peaks, and each fraction was subjected to the insect toxicity tests as described above. Fractions that showed insect toxicity were further separated by a reversed-phase C_18_ column (Everest C_18_, 1.0 mm ID × 250 mm, Grace Vydac). The column was eluted with solvent C and D at a flow rate of 0.05 mL/min using a linear gradient from 20 to 50% solvent D for 45 min (fraction I), from 15 to 60% solvent D for 45 min(fraction II), and from 20 to 40% solvent D for 45 min (fraction III). Chromatographic peaks obtained from each fraction were subjected to insect toxicity tests to find the toxic component. The monoisotopic molecular mass of these components was obtained by LC/MS analysis as described above. 

### 4.5. Determination of N-terminal Sequence

The peptide (200 pmol) was dissolved in the 0.2 M Tris (pH 8.0) buffer (30 μL) containing 6 M guanidine-HCl. To the solution was added 10 μL of 45 mM dithiothreitol (DTT) that was incubated for 1 h at 50 °C. Then, the reaction mixture was mixed with 10 μL of 100 mM iodoacetic acid to alkylate Cys side chains and incubated for 1 h at 28 °C in the dark. The peptide with carboxymethylated Cys residues was purified by HPLC and subjected to Edman sequencing analysis (PPSQ-21A, Shimadzu). 

### 4.6. Enzymatic Digestion

For Lys-C digestion, the peptide solution after carboxymethylation reaction was diluted with a twofold volume of distilled water, which was mixed with Lys-C (Wako Pure Chemical Industries, Osaka, Japan) in an enzyme to a substrate ratio of 1:100 (*w*/*w*). After incubation for 18 h at 37 °C, digested peptide fragments were subjected to HPLC separation on a C_18_ column (TSK-GEL, 1.0 mm ID × 150 mm, TOSOH) eluted using a linear gradient from 15 to 60% solvent D in solvent C for 45 min at a flow rate of 0.05 mL/min. For chymotrypsin digestion, the peptide solution after carboxymethylation reaction was purified by HPLC on a C_18_ column (Everest C_18_, 1.0 mm ID × 250 mm, Grace Vydac) eluted using a linear gradient from 5 to 90% solvent D in solvent C for 85 min at a flow rate of 0.05 mL/min. After lyophilization, the purified peptide was dissolved in 100 μL of distilled water and mixed with chymotrypsin (Roche Diagnostics K.K., Tokyo, Japan) in an enzyme to a substrate ratio of 1:100 (*w*/*w*). After incubation for 18 h at 37 °C, digested peptide fragments were subjected to HPLC separation on a C_18_ column (Everest C_18_, 1.0 mm ID × 250 mm, Grace Vydac) eluted with solvent A and B at a flow rate of 0.05 mL/min using a linear gradient from 5 to 90% solvent B for 85 min. For sequential digestion with Lys-C and chymotrypsin, the peptide solution after Lys-C digestion for 18 h at 37 °C was mixed with chymotrypsin. After incubation for 18 h at 37 °C, digested peptide fragments were subjected to HPLC separation on a C_18_ column as described above.

### 4.7. Homology Modeling

To construct the three-dimensional model, homology modeling software Isolated-FAMS (In-Silico Sciences Inc., Tokyo, Japan) was used [44]. The primary sequence of Bl-1 was automatically aligned with that of Lqh3, and the structure of each toxin was optimized by simulated annealing method of FAMS-ligand using the coordinate of Lqh3 (PDB ID: 1FH3) as a template.

## Figures and Tables

**Table 1:** Insecticidal peptides isolated from the *B. leptochelys* venom.

Name	N-Terminal Sequence (U = Unknown)	Similar Peptides	Toxin Classification
Bl-1	ARDGYISQPENCVYHCFPGS	Lqh3, Bom3	α-like insect and mammal toxin
Bl-2	URDGYLVDDUNCTFFCG	Lqh2, AaH2	α-mammal toxin
Bl-3	UVRDAYIADDKNCVYTCASN	OD1, Bu1	α-insect and mammal toxin
Bl-4	UKNGYAVDSSGKAPECILSNYCNNECTKV	AaHIT1, LqqIT1	β-insect toxin

## Supplementary Materials

The following are available online at https://www.mdpi.com/2072-6651/11/4/236/s1, Table S1. List of monoisotopic molecular masses of the venom components obtained by LC/MS analysis. Numbers shown in bold indicate the peptides isolated in this study. Figure S1. Mass spectra of Bl-1, 2, 3, and 4. Figure S2. Results of LC/MS analysis of native (**A**) and carboxymethylated Bl-1 (**B**). Figure S3. HPLC chromatograms of peptide fragments obtained by degestion with Lys-C (**A**), with chimotrypsin after Lys-C (**B**), and with chymotrypsin (**C**). Figure S4. Product ion spectra of L1 obtained by LC/MS/MS (**A**) and MALDI-TOF/TOF MS analysis (**B**). Product ion spectra of L3 obtained by LC/MS/MS (**C**) and MALDI-TOF/TOF MS analysis (**D**). Figure S5. Product ion spectra of L1 (**A**) and L3 (**B**–**F**) obtained by MALDI-TOF/TOF MS analysis under HE-CID condition. The mass region containing *d-* and *a*- ions necessary for Leu/Ile discrimination was shown. Vertical solid arrows show observed *d*-ions. Figure S6. Product ion spectra of LC1 (**A**) and C1 (**B**) obtained by MALDI-TOF/TOF MS analysis under HE-CID conditions. Figure S7. LC/MS/MS analysis of peptide fragments obtained by chymotrypsin digestion. (**A**) Total ion chromatogram of the fragments. (**B**) Product ion spectrum of C2. (**C**) Fragment ions of C2 observed by LC/MS/MS analysis.
