# Supplementary material for: Isolation and Characterization of Insecticidal Toxins from the Venom of the North African Scorpion, Buthacus leptochelys
Source: Toxins (Basel). 2019 Apr 25;11(4):236. doi: 10.3390/toxins11040236 (PMC6521144; doi:10.3390/toxins11040236)
Supplement: Supplementary file 1 [file toxins-11-00236-s001.pdf]

Supplementary Materials: Isolation and Characterization of Insecticidal Toxins from the Venom of the North African Scorpion, *Buthacus leptochelys*

Yusuke Yoshimoto, Masahiro Miyashita, Mohammed Abdel-Wahab, Moustafa Sarhan, Yoshiaki Nakagawa and Hisashi Miyagawa

Table S1. List of monoisotopic molecular masses of the venom components obtained by LC/MS analysis. Numbers shown in bold indicate the peptides isolated in this study.

MM	MM	MM	MM	MM
500.1	1386.6	2342.0	3919.8	6995.0
515.9	1443.7	2352.0	3945.8	7017.7
524.0	1456.0	2376.0	3982.8	7050.9
536.2	1500.2	2437.0	3987.6	**7107.2**
585.3	1515.0	2480.1	3995.7	7137.9
621.3	1548.7	2504.1	4034.8	7163.9
625.1	1552.0	2543.0	4061.8	**7173.8**
649.0	1588.1	2560.0	4065.8	7191.8
772.1	1705.8	2599.1	4075.8	7268.5
804.0	1710.7	2626.3	4100.7	**7343.9**
858.4	1764.9	2663.1	4101.9	7374.1
873.9	1774.8	2666.1	4183.0	7470.6
905.5	1801.9	2690.1	4192.9	7600.2
914.5	1816.8	2758.1	4196.8	7782.2
950.5	1840.8	2924.2	4229.0	7785.0
954.5	1855.8	3001.2	4232.9	**7828.1**
959.5	1978.9	3082.2	4312.9	7844.2
1007.6	1984.9	3166.3	4364.8	8003.2
1024.1	1998.9	3180.2	4369.1	8025.0
1059.4	2008.0	3164.2	4393.8	8038.2
1066.0	2140.9	3180.2	4394.8	11112.6
1078.5	2154.9	3216.6	4543.1	
1083.5	2164.9	3294.4	4587.0	
1140.6	2179.9	3380.5	5092.7	
1179.7	2189.9	3422.6	5106.2	
1196.7	2225.9	3428.8	5144.2	
1224.6	2303.0	3430.6	5640.5	
1317.6	2304.0	3749.7	6142.5	
1318.6	2327.0	3809.7	6237.8	
1341.6	2338.9	3873.7	6757.0	

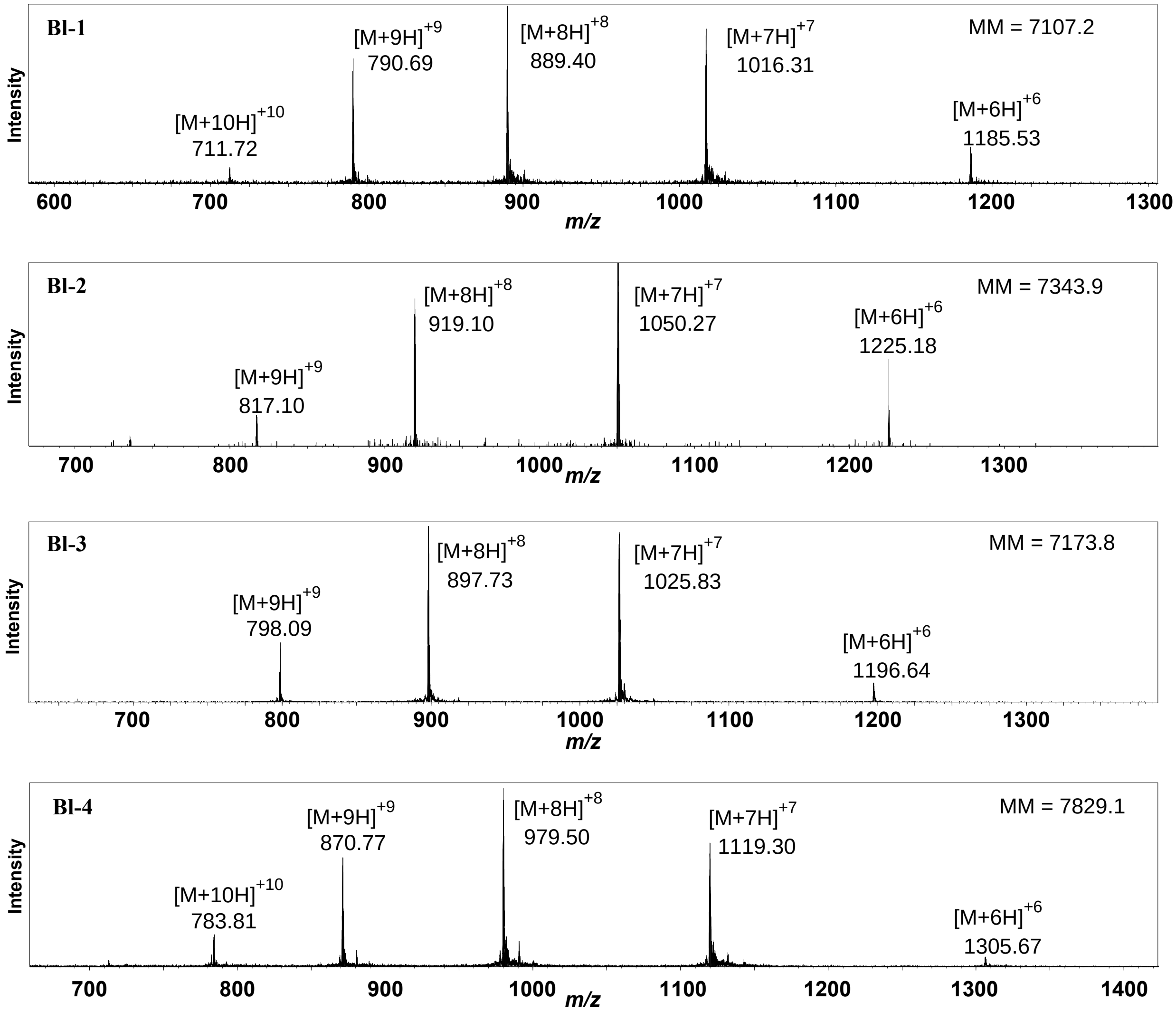

Figure S1. Mass spectra of Bl-1, 2, 3, and 4.

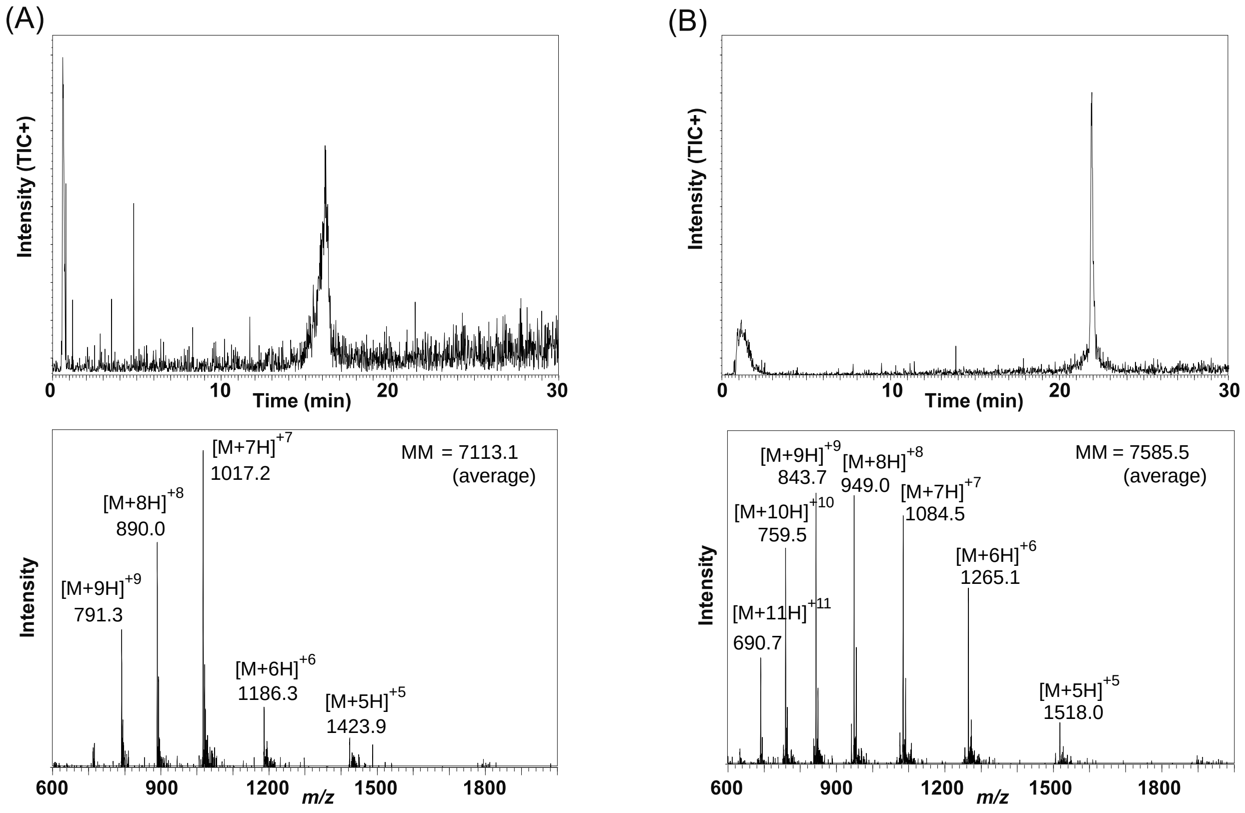

Figure S2. Results of LC/MS analysis of native (**A**) and carboxymethylated Bl-1 (**B**).

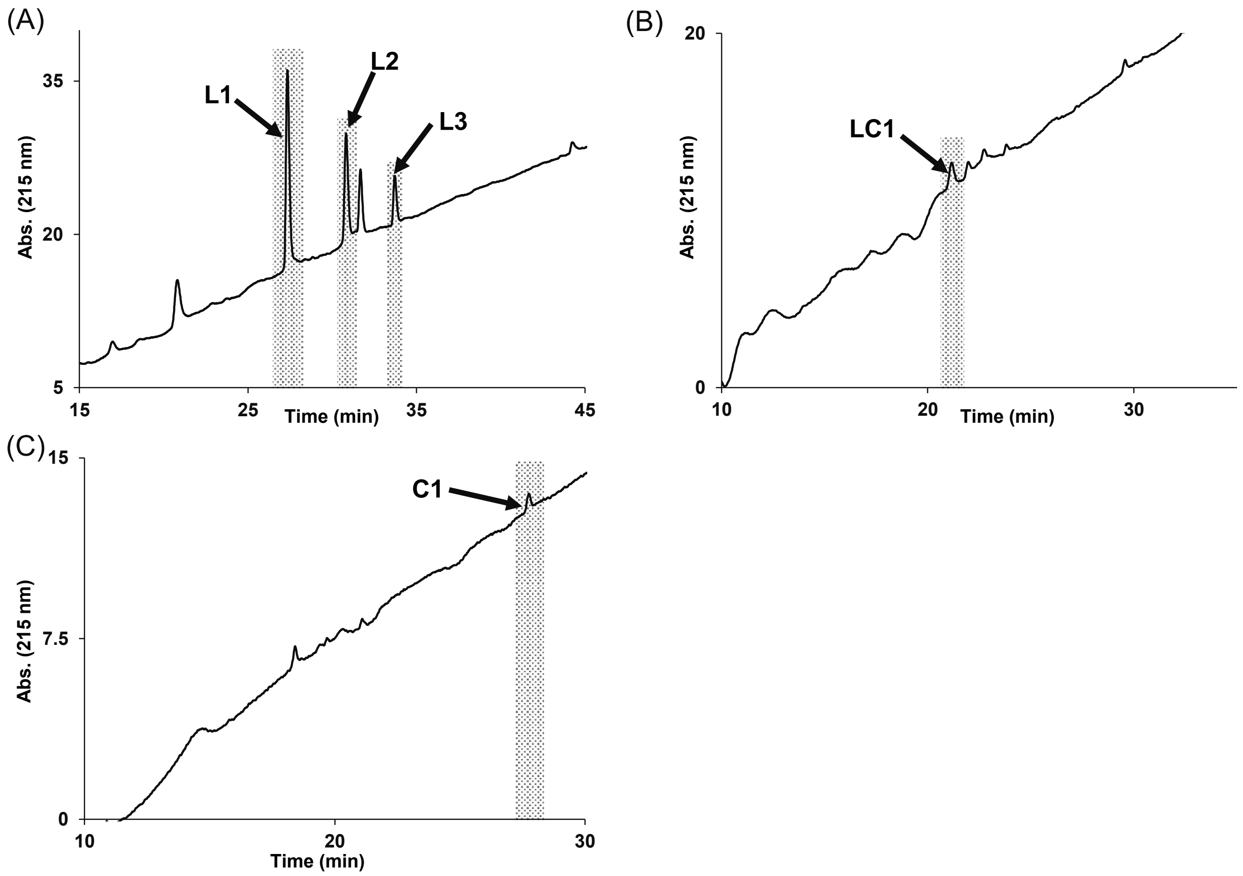

Figure S3. HPLC chromatograms of peptide fragments obtained by degestion with Lys-C (**A**), with chimotrypsin after Lys-C (**B**), and with chymotrypsin (**C**).

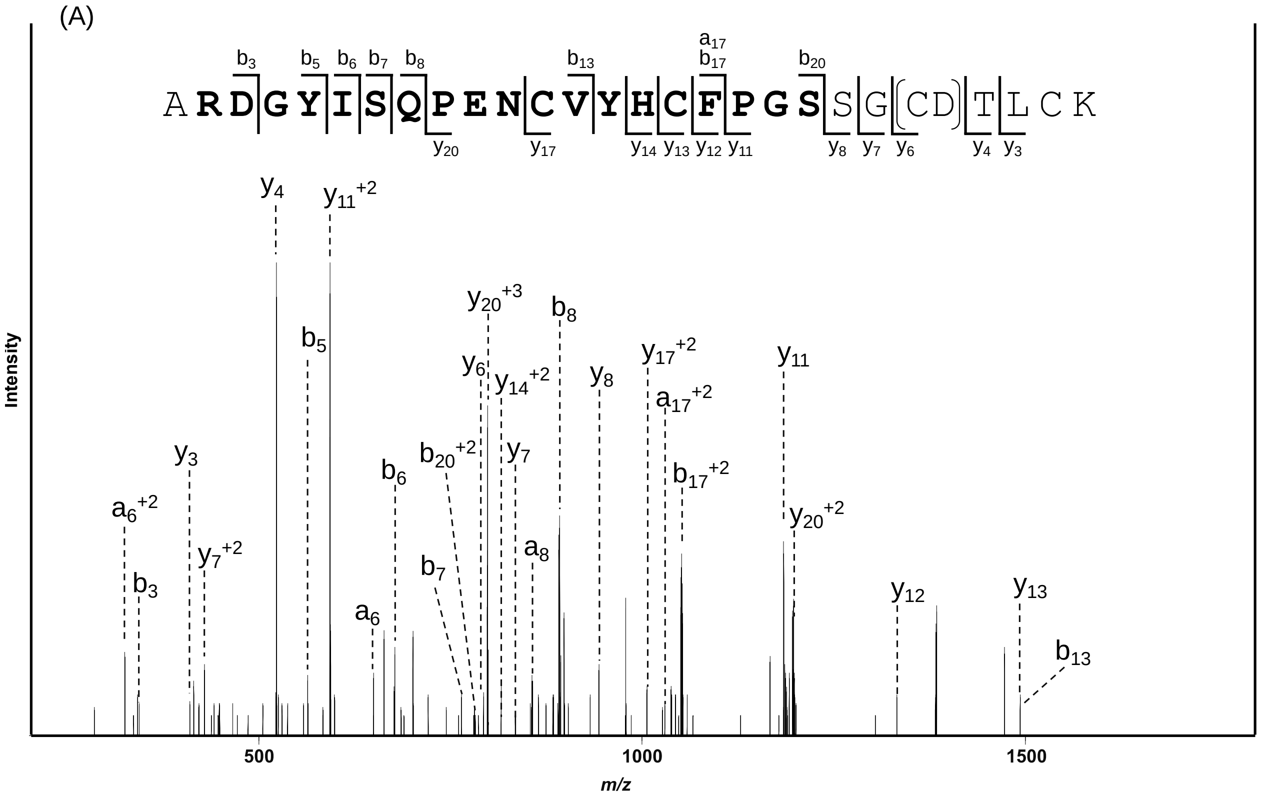
(A)
ARDGYISQPENCVYHCFPGSSGCDTLCK
Intensity
m/z
500
1000
1500

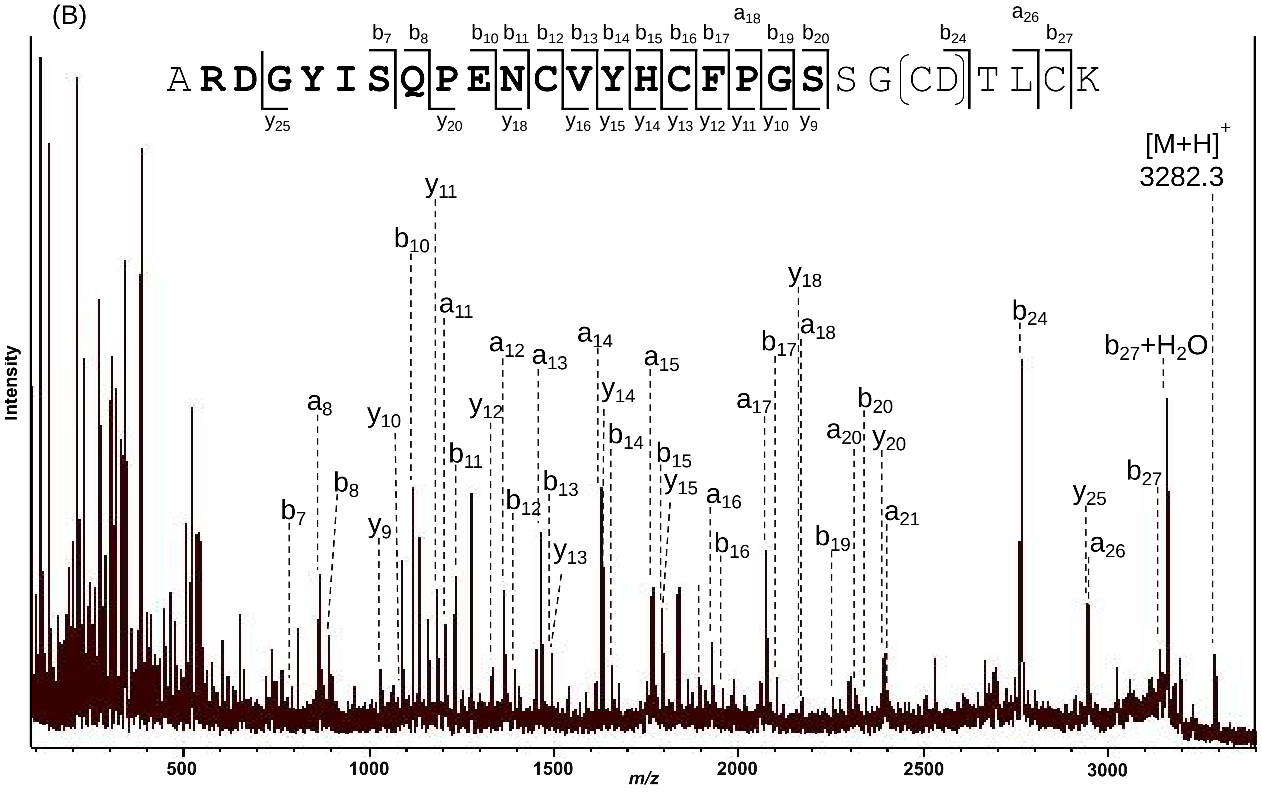
(B)
ARDGYISQPENCVYHCFPGSSGCDTLCK
[M+H]+
3282.3
Intensity
m/z
500
1000
1500
2000
2500
3000

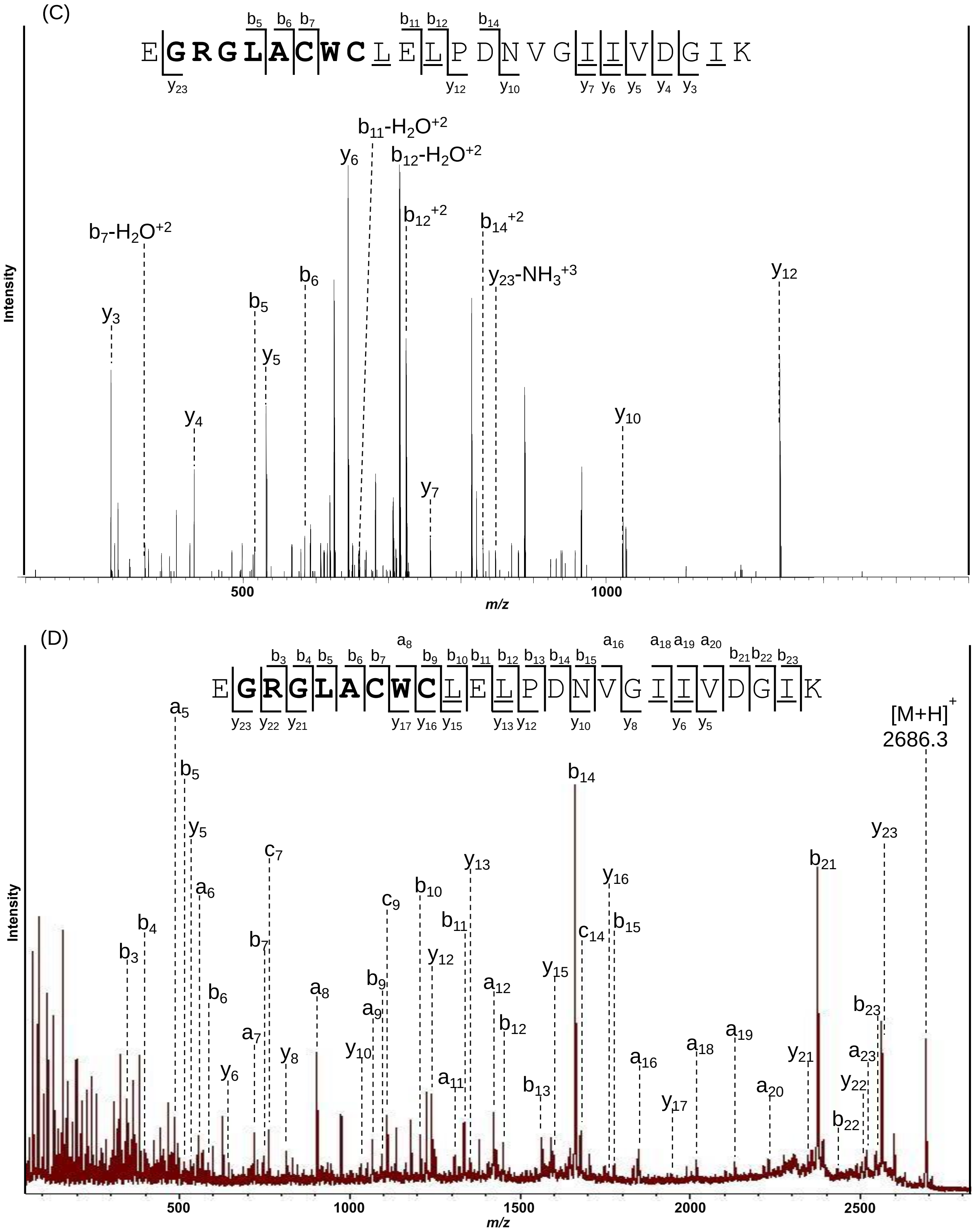

Figure S4. Product ion spectra of L1 obtained by LC/MS/MS (**A**) and MALDI-TOF/TOF MS analysis (**B**). Product ion spectra of L3 obtained by LC/MS/MS (**C**) and MALDI-TOF/TOF MS analysis (**D**).

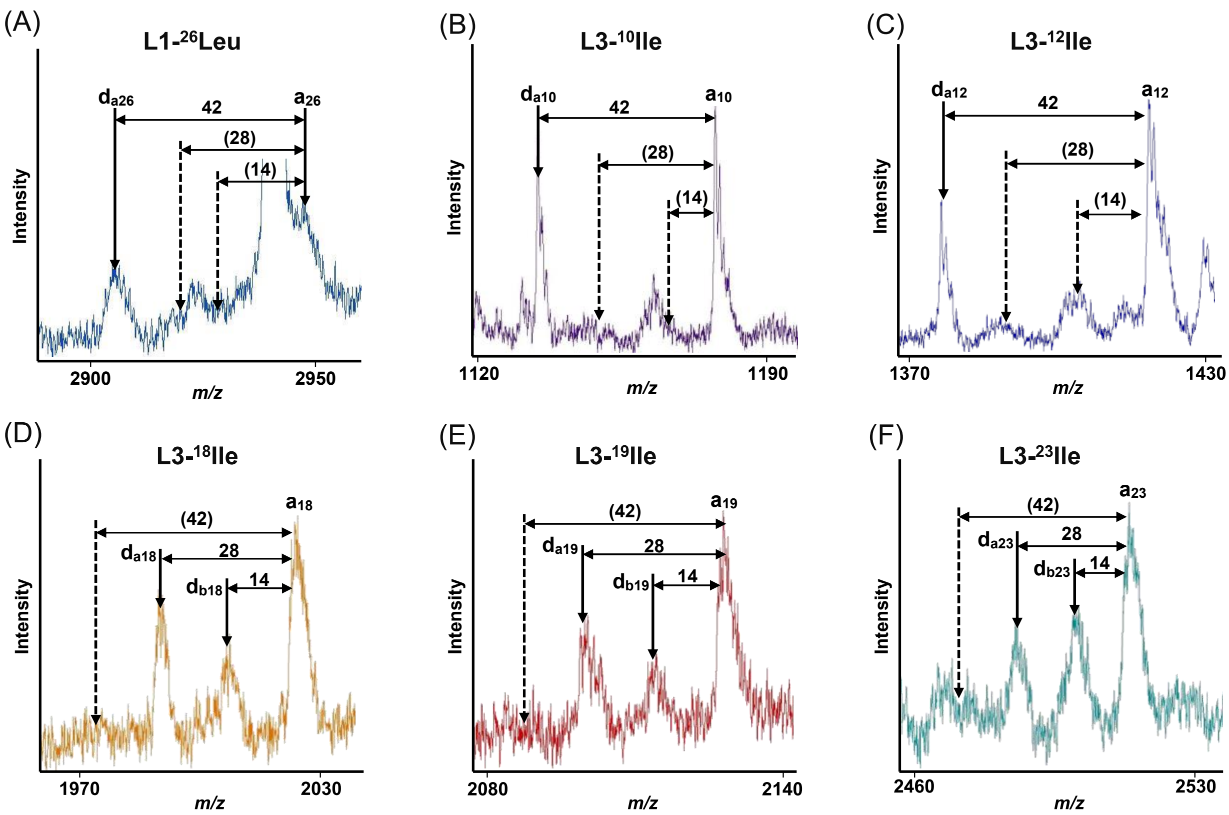

Figure S5. Product ion spectra of L1 (**A**) and L3 (**B**–**F**) obtained by MALDI-TOF/TOF MS analysis under HE-CID condition. The mass region containing *d*- and *a*- ions necessary for Leu/Ile discrimination was shown. Vertical solid arrows show observed *d*-ions.

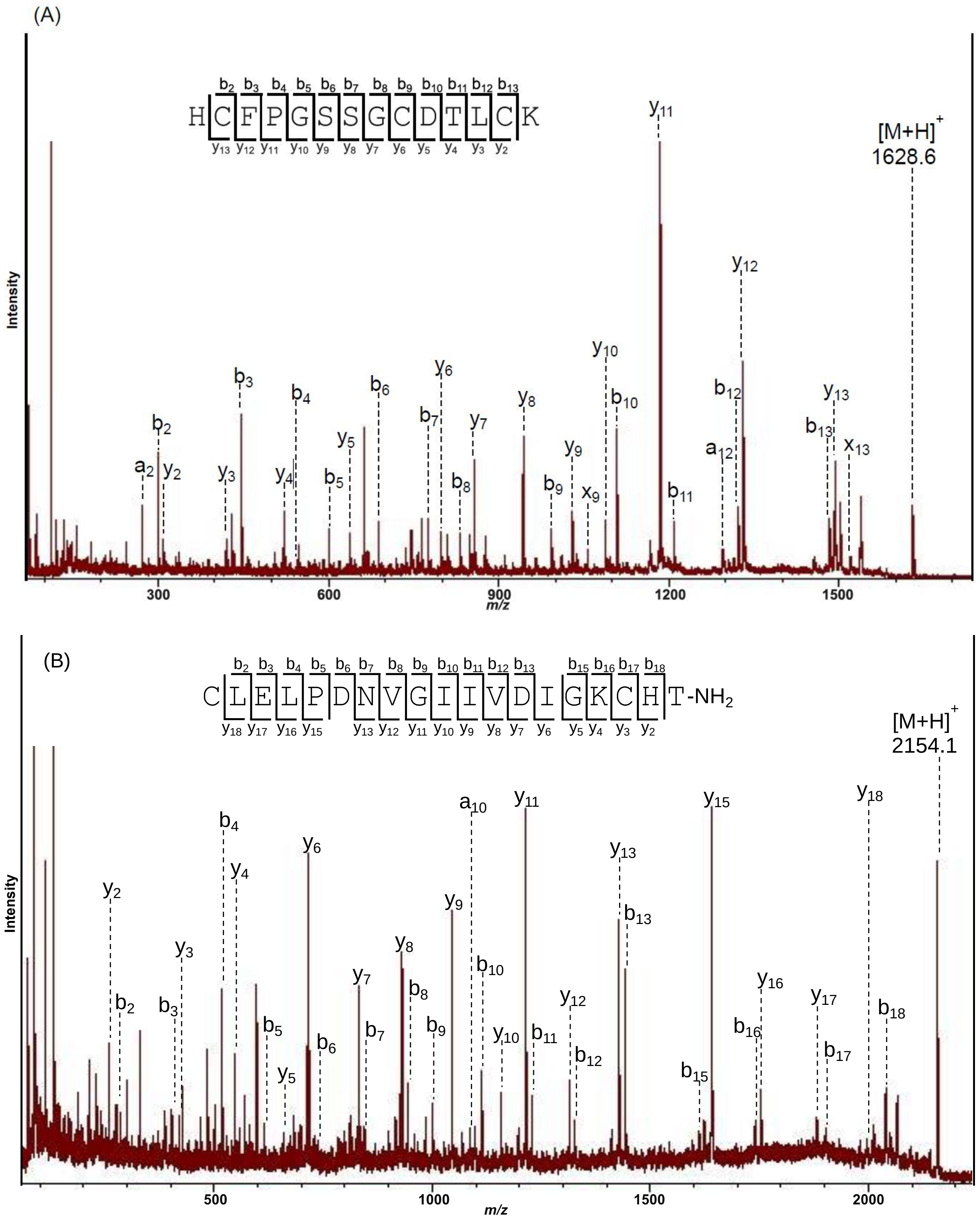

Figure S6. Product ion spectra of LC1 (**A**) and C1 (**B**) obtained by MALDI-TOF/TOF MS analysis under HE-CID conditions.

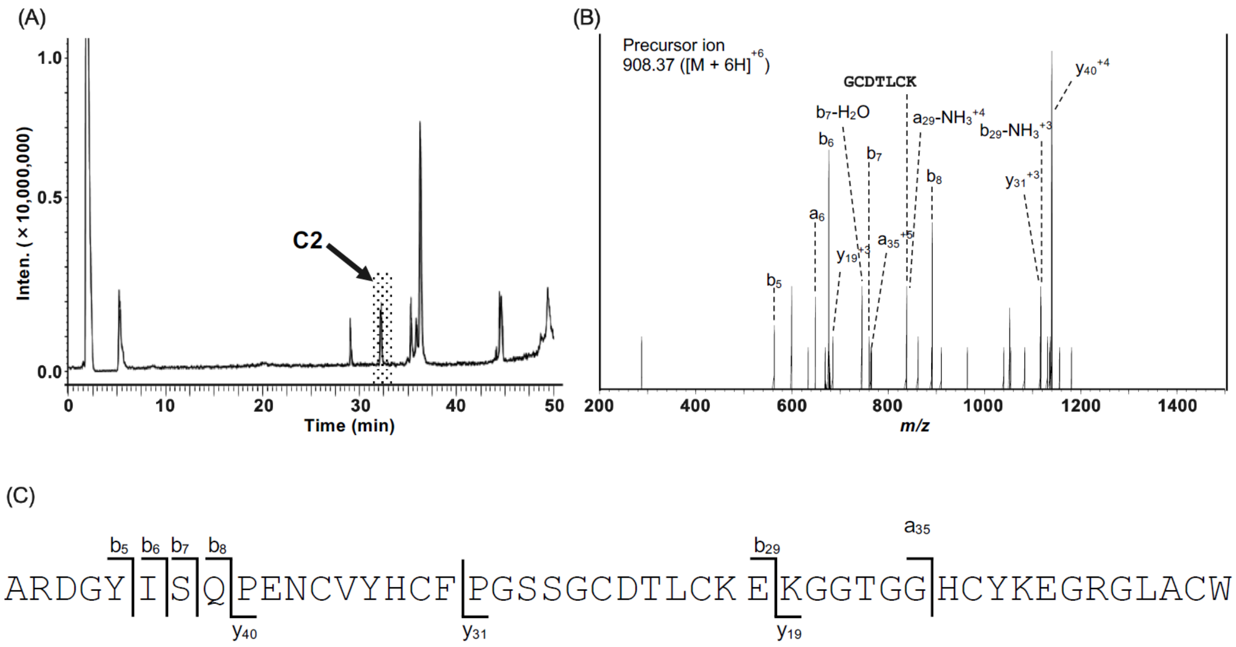

Figure S7. LC/MS/MS analysis of peptide fragments obtained by chymotrypsin digestion. (**A**) Total ion chromatogram of the fragments. (**B**) Product ion spectrum of C2. (**C**) Fragment ions of C2 observed by LC/MS/MS analysis.
